# Complex patterns of signalling to convey different social goals of sex in bonobos, *Pan paniscus*

**DOI:** 10.1038/srep16135

**Published:** 2015-11-05

**Authors:** Emilie Genty, Christof Neumann, Klaus Zuberbühler

**Affiliations:** 1 Department of Comparative Cognition, Institute of Biology, University of Neuchâtel, rue Emile Argand 11, 2000 Neuchâtel, Switzerland; 2 School of Psychology and Neuroscience, University of St Andrews, St Andrews, KY16 9JP, Scotland (UK)

## Abstract

Sexual behaviour in bonobos (*Pan paniscus*) functions beyond mere reproduction to mediate social interactions and relationships. In this study, we assessed the signalling behaviour in relation to four social goals of sex in this species: appeasement after conflict, tension reduction, social bonding and reproduction. Overall, sexual behaviour was strongly decoupled from its ancestral reproductive function with habitual use in the social domain, which was accompanied by a corresponding complexity in communication behaviour. We found that signalling behaviour varied systematically depending on the initiator’s goals and gender. Although all gestures and vocalisations were part of the species-typical communication repertoire, they were often combined and produced flexibly. Generally, gestures and multi-modal combinations were more flexibly used to communicate a goal than vocalisations. There was no clear relation between signalling behaviour and success of sexual initiations, suggesting that communication was primarily used to indicate the signaller’s intention, and not to influence a recipient’s willingness to interact sexually. We discuss these findings in light of the larger question of what may have caused, in humans, the evolutionary transition from primate-like communication to language.

In most mammalian species, sexual interactions are typically restricted to a limited time period of female fecundity^1^,^2^. In bonobos (*Pan paniscus*) and to a lesser extent chimpanzees (*P. troglodytes*), however, sex occurs frequently for social functions that go beyond mere reproduction, including paternity confusion, exchange of benefits, and, for bonobos, mediation of social relationships^2^,^3^,^4^,^5^. This peculiar functional diversification of sex in bonobos seems to be linked to their female-centred, egalitarian social structure and to the fact that the receptive state of females is extended beyond the ovulation period, which is possibly also responsible for low inter-male sexual competition, high intra- and inter-group tolerance^6^,^7^,^8^,^9^, and high social status of females^2^,^5^,^10^,^11^,^12^,^13^.

From an early age, bonobos engage in sexual interactions in almost every partner combination, which includes heterosexual mounting with penis insertion (copulation), homo- and hetero-sexual mounting without penis insertion (pseudo-copulation) and female homo-sexual genital rubbing^7^,^14^,^15^. Sex can be used to reduce tensions^3^,^16^,^17^,^18^,^19^,^20^,^21^, especially when competing over food^3^,^4^,^18^,^22^ where it is offered to gain access to resources^3^,^7^,^16^, following aggression as a form of consolation from bystanders^20^, or between opponents as reconciliation^23^,^24^. Reconciliation sex between opponents is almost always in the form of mounting or genital touching, whereas consolation can also involve non-sexual contacts, such as embraces^21^. Here, bystanders may lower their own risk of suffering from redirected aggression^19^ in addition to reducing the distress of a close social partner^17^,^20^. A third major social function of sex in bonobos is to facilitate the formation of female social bonds^2^,^25^,^26^, which appears to be instrumental in allowing females to coexist and co-feed peacefully, to form strong coalitions and to exert social power^7^,^11^,^15^,^22^,^24^.

## Communication during sexual solicitations

**Figure 1:**
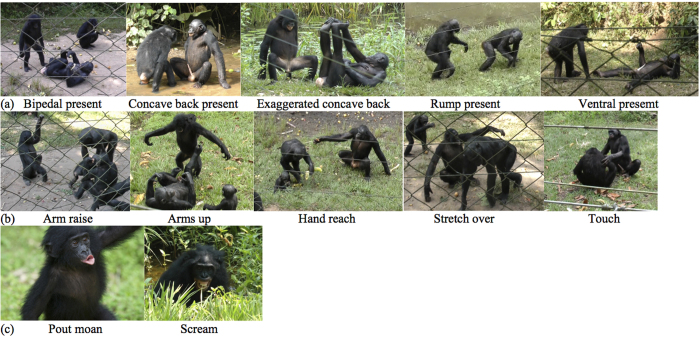
Communicative signals used during sexual solicitations. (**a**) sexual initiation postures, (**b**) most commonly used gestures, (**c**) facial expressions accompanying the most commonly used vocalisations.

The default way to initiate sex in this species is to approach and solicit a partner with ‘genital offers’, i.e. specific sexual initiation postures including ‘concave back present’, ‘rump present’, or ‘ventral present’^27^ (see Fig. 1a). Sometimes, however, genital offers are accompanied by body movements (e.g. swaying the upper body), facial expressions (e.g. silent bared-teeth), vocalisations (e.g. pout moan, scream) and gestures (e.g. hand-reach, touch, stretch over, beckoning, branch shaking)^3^,^26^,^27^,^28^,^29^,^30^,^31^,^32^. An early study conducted on three captive bonobos demonstrated that gestures were used to position the partner for sex and that specific gestures lead to specific positions^28^. The communication behaviours described above do not appear before early adolescence despite the fact that copulation-like genital contacts and sex for tension reduction in the presence of food already occur from a very early age^33^,^34^,^35^ without clear signs of gradual learning through exposure to adult sex^33^,^34^ and with no known equivalent in any other great ape species^33^,^34^.

## Aims and predictions

In this study, we focused on the communication behaviour to solicit sex in various social contexts (appeasement, social bonding, tension reduction and reproduction). We were interested in whether signalling differed according to the social goal and the gender of the initiator and whether signals differed in how successful they were in persuading a partner to have sex. We only considered the signals that qualified as intentional, following the definition used in previous studies of animal communication^36^,^37^,^38^,^39^,^40^. For a signal (gestural or vocal) to qualify as intentional, the signaller (1) produces the signal only in the presence of an audience while orienting its body and/or gaze to a specific recipient, (2) engages in audience checking (looks at the targeted recipient before or during signalling and/or alternates gaze between the recipient and an event or object), specifically for gestural signals (3) adjusts the signal according to the visual attention of the recipient (uses silent signals only if recipient is fully attending, produces attention getters to inattentive recipients, i.e., uses audible or tactile signals, or changes location to face recipient), (4) shows response waiting (pauses and maintains visual contact with recipient after signalling) if the goal is not met immediately and (5) shows persistence (repeats the same signal) or elaboration (uses new signal or combination of signals) in case of failure^36^,^37^,^38^,^39^,^40^.

Bonobos produce a wide range of signals before interacting sexually with a partner. One study suggested that gestures were used to physically position the partner for sex^28^. Since sex is used for various social goals in this species, an alternative hypothesis is that signal production is used to express the social goal of the signaller. To address this possibility, we tested the effects of social goal and gender on signal categories (gestures, vocalisations, multi-modal combinations and sequences of signals) and signal types (specific gestures and vocalisations). We predicted that the communicative signals produced in addition to the sexual initiation postures were specific to the intended social goal.

A second hypothesis is that some types of signals are more successful in persuading potentially reluctant recipients to have sex than others. To address this, we tested whether initiation success (initiations that led to sexual interactions) was related to signal categories (gestures, vocalisations, multi-modal combinations and sequences of signals), social goal, and gender. We predicted that the likelihood of successful initiations depended on the type of signals used.

## Methods

### Ethics statement

The experimental protocol for this study was performed in accordance with the approved ethical ASAB/ABS Guidelines for the Use of Animals in Research and was conducted in compliance with animal care regulations and applicable national laws (research permit: MIN.RS/SG/004/2009). The experimental protocol was also approved by the scientific coordinator and scientific committee of ‘Les Amis des Bonobos’ for this study.

### Study groups

We collected data from two social groups at the ‘Lola Ya Bonobo’ sanctuary, DRC, between February and June 2012. During the study period, group 1 consisted of 22 individuals, including adult, subadult and juvenile males and females and 1 infant. Group 2 consisted of 20 individuals with adult, subadult and juvenile males and females, and 1 infant (age classes as defined by^7^).

Both groups live in two large forested enclosures of 10 and 15 ha, respectively, composed of patches of primary rainforest, lakes, swamps, streams, and open grassy areas. In this semi-natural environment, individuals exhibit a large range of behaviours also observed in the wild^41^. During the day, the bonobos can move freely, forage for wild fruits, leaves, and herbaceous vegetation in the forested parts of their enclosures, in addition to three feedings provided by caregivers. The feeding routine is to distribute fruits in the morning, to give a mixture of soya milk (supplemented with milk, maize, honey and nutriments) around midday, and to distribute vegetables in the afternoon. Each day, caregivers distribute approximately 6 kg of fruits and vegetables to each individual. The bonobos are also provided with daily supplemental feeds comprising of seasonal fruits and nuts. Water is freely available from lakes, ponds and streams within their enclosures, with fresh water (with added salt and sugar) additionally distributed several times a week. At night, all individuals are kept in dormitories of approximately 75 m^2^, divided in several separable rooms and connected to the outside enclosures by a tunnel.

### Data collection and analysis

Observations took place over 68 days, and included 222 hours of observation time, split equally between the two groups. Observations usually started around 08.30am and continued through mid-afternoon. As all the observations were done in association with feeding times, all members of the group were visible or present at the edge of the forest. Behavioural data were collected using all-occurrence sampling^42^ with a focus on how sexual social interactions were initiated and communication behaviour was deployed.

We used Filemaker Pro to administer the resulting database. Social interactions were recorded with a Panasonic HD digital camcorder (HDC-SD900) equipped with a directional microphone (Sennheiser MKE 400).

For subsequent analysis, we only considered sexual solicitations events initiated by adult, subadult and late juvenile individuals. We focused our analysis on the communicative signals (gestures and/or vocalisations) that occurred in addition to the sexual initiation postures (Fig. 1a). Data were divided between female and male initiators because some social goals were gender-specific, i.e. social bonding was restricted to female-female interactions while “true” copulations were almost exclusively initiated by males as demonstrated in previous studies^7^,^8^,^26^. Therefore, female sexual initiations were tested for the following social goals: appeasement, tension reduction and social bonding and male initiations for appeasement, tension reduction and reproduction.

### Coding of sexual solicitations

For each observed sexual initiation, we coded the (a) identity, sex and age class of signaller and recipient (as identified by the orientation of the signaller), (b) their relative rank (signaller higher, equal or lower than recipient) (c) recipient’s attentional state (fully attending, head direction 45 ° to 90 ° from signaller, or not attending), (d) distance between signaller and recipient, (e) observed social goal of sexual initiation (see definition section), (f) type of sequence (uni- or multi-modal) (see definition section), (g) duration of signalling sequences (s), (h) type of sexual initiation posture (Fig. 1a, definition section), (i) type of gesture (see definition section, Fig. 1b) (j) modality (silent, auditory, contact) of gestures, (k) type of vocalisation (see definition section, Fig. 1c), (l) recipient reaction, (m) presence or absence of response waiting, (n) presence or absence of persistence (repetition of signal and/or elaboration), (o) success or failure of interaction.

## Definitions

### Observed social goal of sex

#### Reproduction

No fight or threat just before, no on-going play, direct approach from signaller, persistence in reaching true copulation. Additional criteria: evidence for ejaculation, female at maximum swelling, courtship from several males, signaller (and potentially other males) follows female around and repeats initiation, attempt to lead female away from the rest of the group^32^, female initiation, copulation follows initiation. Can only be heterosexual.

#### Tension reduction

competitive context in the presence of food or other desirable item or male-male competition for access to female, no fight or threat just before, no on-going play, careful approach from signaller. Optional criteria: accompanied by general group arousal, clear attempt to gain access to food resource. Can be hetero- or homosexual.

#### Appeasement

Fight or threat occurred just before. Optional criteria: signs of distress disappear following sexual contact. Can be reconciliation between opponents or consolation from third party. Can be hetero- or homosexual.

#### Social bonding

No fight or threat just before, no food or other desirable item is present, no on-going play. Can only be homosexual (female-female).

### Sequences and multi-modal combinations

Sequences were defined as strings of two or more signals made by the same individual within less than 1s of each other. Multi-modal combinations were defined as a combination of two or more signals of different sensory modalities (i.e. call and gesture) produced within less than 1s of each other. If inter-signal intervals surpassed 1s, we considered them as belonging to separate sequences. This criterion had previously been arbitrarily established and used in gestural research and we thus decided to apply it to make our study comparable with previous work^43^,^44^,^45^. Strings of two or more sequences by the same individual were defined as a communicative bout (as per^45^).

### Communicative signal types

#### Sexual initiation postures (Fig. 1a)

##### Bipedal present

Standing bipedally in front of recipient with arms spread apart

##### Concave back present

Sitting in front of recipient with arched back to expose genitals with legs spread apart.

##### Exaggerated concave back present

Standing quadrupedally in front of recipient with ventral side up to expose genitals with legs spread apart.

##### Rump present

Standing quadrupedally in front of recipient with dorsal side up to expose hindquarters, while looking back at recipient.

##### Ventral present

Lying on back in front of recipient with legs spread apart to expose genitals.

#### Gestures (most commonly used during sexual initiations: see statistical analyses section) (Fig. 1b)

##### Arm raise. 

 Raising one arm above the head, visual silent.

##### Arm(s) up

Raising one or both arms laterally on side of body, visual silent.

##### Hand reach

Extending arm and hand towards another individual, visual silent.

##### Stretch over

Raising stretched arm to head level above recipient’s body, palm facing down with bent wrist, visual silent.

##### Touch

Touching gently another individual’s body part with palm of hand, tactile.

#### Vocalisations (most commonly used during sexual initiations: statistical analyses section) (Fig. 1c)

##### Pout moan

Low-pitched, melodious call sounding like a whining “hoo-hoo”^27^, accompanied by pout face (lips are pursed forward and curled outward in front resulting in circular opening).

##### Scream

High-pitched, with shrill and rasping sounds given at full vocal strength, large number of harmonics and long in duration, accompanied by teeth-baring (complete lip retraction, exposing both teeth and gums, the mouth may be wide open)^27^.

### Statistical analysis

We built generalized linear mixed models with binomial error structure and logit link function^46^,^47^ to investigate (1) differences between social goals in usage of general signal types (gestures, vocalisations, multi-modal combinations or sequences of signals) (models 1a and 1b) and (2) differences between social goals in how general signal types influenced whether initiations were successful or not (models 2a and 2b). We built separate models for female and male initiators. The response variable in models 1a and 1b was whether or not a given signal type occurred during an initiation. Specifically, we created four lines per initiation (corresponding to the four investigated signal types) and scored, separately for each signal type, 1 if the signal type occurred and 0 if the signal type did not occur. In models 2a and 2b, the response variable was whether or not an initiation was successful. In the initial models we included the interactions between social goal and signal types and removed interactions if they did not improve model fit as assessed by likelihood ratio tests^48^. In all models, we used signaller age, receiver age and sex, and spatial distance between the two interacting individuals at the beginning of an interaction as control predictor variables. Random effects were signaller and receiver identity and for models 1a and 1b the initiation number to control for the repeated inclusion of each initiation (see above). We treated these models as our full models (i.e. models that included all predictor variables) as opposed to null models, which included the control predictor variables and the random effects. All models were fitted with the glmer function of the lme4 package (version 1.1–7)^49^ in R 3.1.1^50^. To compare overall success rates between males and females, we used a non-parametric Mann-Whitney-*U* test. More details on the analysis can be found the supplementary material. The analyses were conducted on a data set of n = 292 events initiated by n = 17 females and n = 719 events initiated by n = 19 males.

We then conducted post-hoc descriptive analyses to further assess whether specific communicative signals (gestures and/or vocalisations) were more likely to be produced for the different goals of sex in order to clarify the signaller’s intention. We restricted our analysis to the most commonly used signals during sexual initiations. To be considered common, the signals had to occur in at least 10% of initiations in at least one sex-goal combination. Two vocalisations (‘pout moan’ and ‘scream’) and five gestures (‘arm raise’, ‘arm up’, ‘hand reach’, ‘stretch over’, ‘touch’) met our criterion and were selected for subsequent analysis. We calculated the proportions of cases with which each of the seven signals had been used at least once in an initiation for each of the social goals. We restricted the analysis to those individuals for which at least five initiations had been observed for a given function and present mean proportions per signal/goal, weighted by the respective number of initiations for each contributing individual. These analyses were conducted on a data set of n = 959 events initiated by n = 12 females and n = 17 males.

## Results

### I. Social goal and gender effects on signal production (models 1a and 1b)

#### Use of signal categories

**Figure 2:**
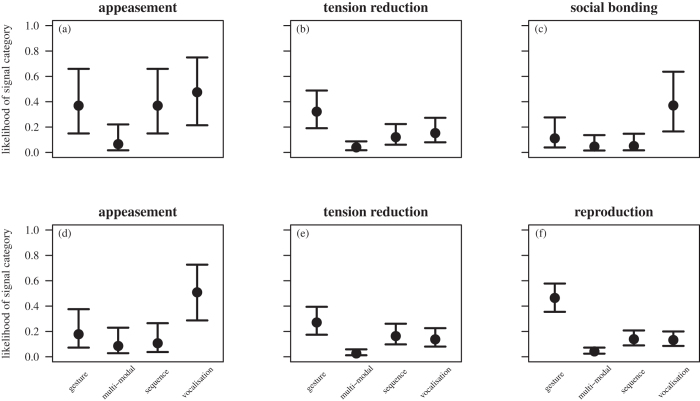
Production of signal categories (gestures, vocalisations, multi-modal combinations and sequences) for females (top row) during (a) appeasement, (b) tension reduction and (c) social bonding interactions and males (bottom row) during (d) appeasement, (e) tension reduction and (f) reproduction interactions. Shown are model estimates alongside standard errors.

We first tested the effect of social goal and gender on how individuals used the different signal categories (gestures, vocalisations, multi-modal combinations and sequences of signals). We found that the full models differed significantly from their respective null models (females: chi^2^ = 136.94, df = 11, P < 0.0001; males: chi^2^ = 344.61, df = 11, P < 0.0001), with both female and male initiators using the different signal categories depending on the specific goal (females: chi^2^ = 37.78, df = 6, P < 0.0001; males: chi^2^ = 43.93, df = 6, P < 0.0001; Tables 1 and 2; Fig. 2). In particular, females and males made little use of multi-modal combinations across all social goals but were more likely to use gestures to initiate sex for tension reduction and vocalisations to initiate sex for appeasement. Females were more likely to use gestures to initiate sex for appeasement than for social bonding (Fig. 2, top row) and more likely to use vocalisations to initiate sex for appeasement and social bonding than for tension reduction. Females also used sequences of signals predominantly to initiate sex for appeasement. Males were more likely to use gestures to initiate reproductive sex than to initiate sex for appeasement (Fig. 2, bottom row).

#### Use of specific signals

**Figure 3:**
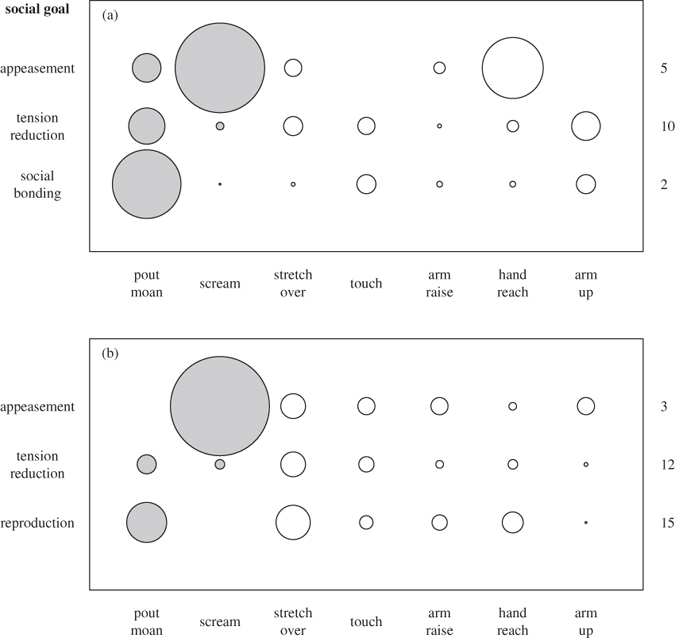
Use of specific vocalisations and gestures for different social goals in (a) females and (b) males. The size of circles is indicative of the average proportion of interactions in which a signal was used for a given function (the largest circles represent 0.47 and 0.52 in (**a**,**b**), respectively). Sample sizes (number of individuals) are given to the right of each goal. Vocal signals are represented by filled circles, gestures by open circles.

When analysing the use of the seven most commonly used signals (5 gestures and 2 vocalisations; see statistical analysis section, Figs 1 and 3), we found that not all individuals produced all signals and that the proportion of signals used within individuals varied across social goals (see supplementary Figs S1 and S2).

Both males and females produced ‘screams’ more frequently to initiate sex for appeasement than for any other goal (Fig. 3a,b). Females produced ‘pout moans’ more frequently to initiate sex to bond socially than for any other goal, while males produced ‘pout moans’ more frequently to initiate reproductive sex than for any of the other social goals (Fig. 3a,b). Females used ‘hand reach’ more frequently to initiate sex for appeasement than for any other goal (Fig. 3a). The other four gestures (‘arm raise’, ‘arm up’, ‘stretch over’ and ‘touch’) were not linked to any specific social goal although ‘touch’ and ‘arm up’ were never used to initiate sex for appeasement. Sex for appeasement in females, thus, is reliably initiated by screaming and/or hand reaching. For males, we found no relationship between gestures and specific social goals of sex.

We could not reproduce earlier findings demonstrating that some gestures were used to position the partner either in a ventro-dorsal or ventro-ventral position^28^. However, in Savage Rumbaugh *et al.*’s^28^ study most of the observed gestures were positioning, tactile and mechanically effective gestures and, to our knowledge, initiator gender was not controlled for. In our study, and following the definition of intentional signals, behaviours that were mechanically effective in reaching a goal (sexual interaction) by physical force, were not included in our analyses. We also found that most of male initiations led to ventro-dorsal positions (66.0%) and most of female initiations led to ventro-ventral positions (77.0%).

### II. Signal category, social goal and gender effects on success rates (models 2a and 2b)

#### Gender effects

Overall, initiations were successful in less than half of all interactions with no gender effect. Female initiations were successful in 33.2% of cases, while males were successful in 41.2% of cases (MWU test, W = 163.5, n_females_ = 17, n_males_ = 19, P = 0.962). Both full models differed from their respective null models (females: chi^2^ = 29.11, df = 13, P = 0.0063; males: chi^2^ = 35.83, df = 13, P = 0.0006). None of the tested interactions between social goal and signal categories were significant (males and females), so we removed all interactions from the final models (max chi^2^ = 4.24, df = 2, all P > 0.1201, four tested interactions).

#### Social goal effects

**Figure 4:**
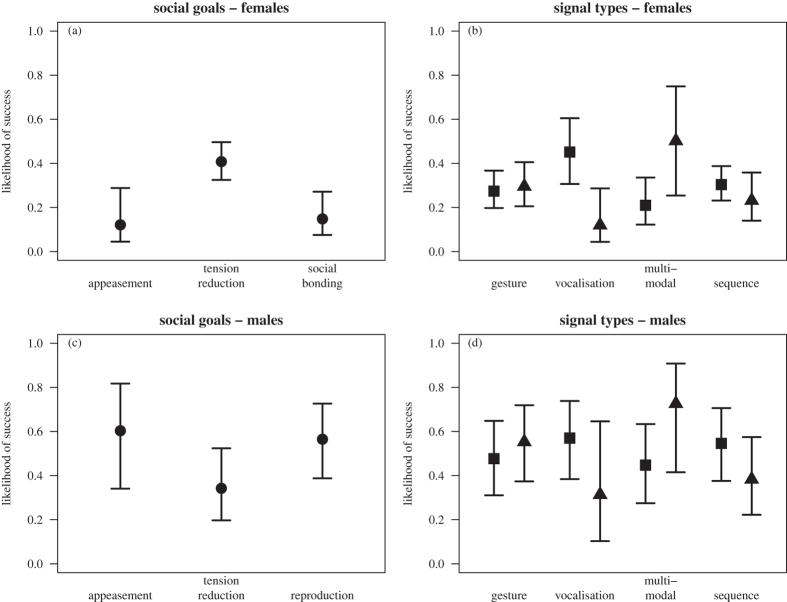
Differences in success depending on social goal (left panel) and general signal categories (right panel) for female (top row a and b) and male (bottom row c and d) initiators. In the right panel, squares indicate absence and triangles indicate presence of a signal category. Shown are model estimates with associated standard errors.

Females were most successful in obtaining sexual interactions in the context of tension reduction (chi^2^ = 15.70, df = 2, P = 0.0004), while the opposite was the case for males (chi^2^ = 14.89, df = 2, P = 0.0006, Fig. 4, Tables 3 and 4).

#### Signal category effects

For both females and males, initiations including vocalisations or signal sequences tended to decrease the likelihood of success (Fig. 4, right panel, Tables 3 and 4). For females, but not for males, vocalisations were significantly associated with a reduced likelihood of success (females: β = -1.80, se = 0.81, P = 0.0260, males: β = -1.07, se = 0.78, P = 0.1709), while for males, but not for females, signal sequences were significantly associated with reduced likelihood of success (males: β = -0.66, se = 0.25, P = 0.0070, females: β = -0.37, se = 0.37, P = 0.3153). On the other hand, initiations including gestures or multi-modal signals were more likely to be successful (Fig. 4, right panel), although this effect was not significant (Gesture, females: β = 0.10, se = 0.34, P = 0.7545; males: β = 0.31, se = 0.22, P = 0.1558; Multi-modal, females: β = 1.33, se = 0.82, P = 0.1045; males: β = 1.19, se = 0.74, P = 0.1111; Fig. 4, Tables 3 and 4).

## Discussion

In this study we assessed how bonobos deployed their species-specific vocal and gestural repertoire to initiate sex. We were interested in whether the choice of signals was related to the four main social goals of sex in this species - appeasement, social bonding, tension reduction and reproduction - and the gender of the initiator and whether signals differed in how successful they were in persuading a partner to have sex.

We did not find clear one-to-one links between specific signalling patterns and specific goals, although a number of general effects emerged. First, vocalisations were generally more function-specific than gestures or multi-modal combinations. Second, gestures were more likely to be used to initiate sex for tension reduction, while vocalisations were more likely used to initiate sex for appeasement. Multi-modal combinations occurred but were generally rare for all goals.

For both males and females, the usage of signal categories differed between goals. Females were more likely to use gestures to initiate sex for appeasement and for tension reduction than for social bonding. ‘Hand reach’ and ‘screams’ were relatively specific to initiations of sex for appeasement, while ‘pout moan’ was relatively specific to sex for social bonding. Females, but not males, also used signal sequences to initiate sex for appeasement. Males were more likely to use gestures to initiate sex for tension reduction and reproduction than for appeasement. For vocalisations, ‘screams’ were more likely to be produced to initiate sex for appeasement than any other social goal, similarly to females, while ‘pout moans’ were most frequently used to initiate reproductive sex. Thus, a remarkable pattern is that male reproduction and female social bonding intentions are advertised with the same vocal behaviour.

Finally, we found that, for both sexes, initiations were generally not very successful, with no clear evidence that certain communicative strategies improved initiation success, suggesting that signalling has more to do with conveying intention to a recipient than persuading him or her to have sex.

### Why do bonobos use a wide range of signals to initiate sex?

**Figure 5:**
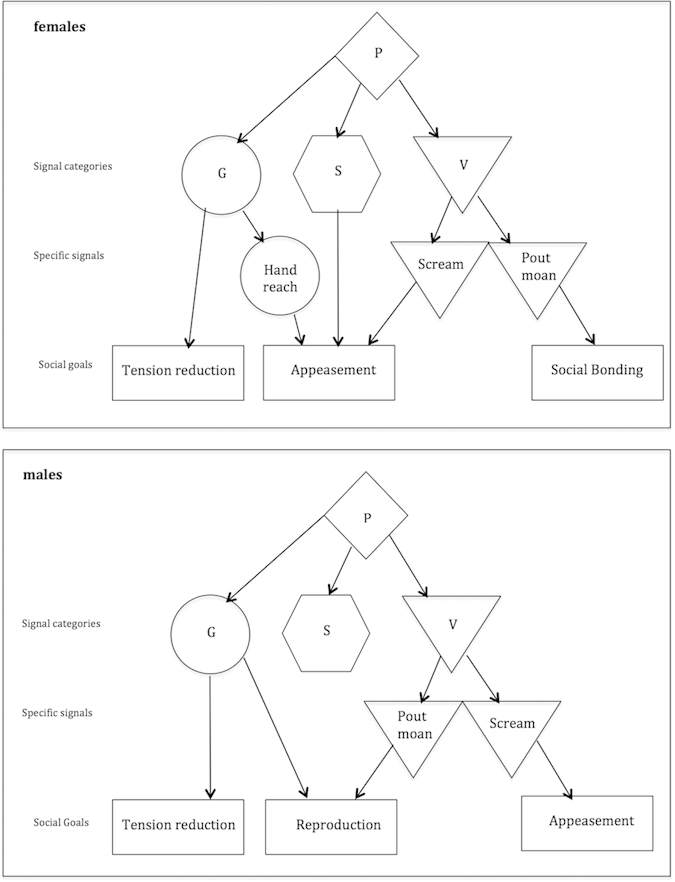
Summary plot of the most likely communicative strategy depending on the social goal of sex and gender of initiator. Arrows represent the most likely scenario based on the result of the statistical models for signal categories and on the descriptive analyses of proportions for specific signals. P = Sexual initiation posture; G = Gesture; S = Sequence; V = Vocalisation.

Our data show that ‘screams’ were specific to sex initiations for appeasement while ‘pout moans’ were specific to sex initiations for social bonding (females) or reproduction (males). For gestures, ‘hand reach’ was specific to sex initiations for appeasement (females only), while sex initiations for tension reduction were linked with all other gestures (males and females), suggesting that tension reduction encompasses a range of situations requiring a range of signalling strategies (Fig. 5). In light of these results it could be argued that ‘screams’ are unintentional reflections of the emotional distress experienced during the aggression preceding appeasement. However, ‘scream’ production was generally maintained until the partner consented to a sexual interaction, upon which it ceased, or in case of unsuccessful initiations, it could persist for long time periods, suggesting that the signaller pursued a specific social goal.

In general, initiations with sexual initiation postures alone or with additional communicative signals were successful less than half of the time and we found no clear relation between communication behaviour and success. Females were more successful in initiating sex for tension reduction compared to other goals, while the opposite was found for males, suggesting that females are better at negotiating sex in competitive situations than males. Gestures and multi-modal signals were associated with higher likelihoods of success, while vocalisations and signal sequences were associated with lower likelihoods of success. Vocalisations reduced the likelihood of success in females, while signal sequences reduced the likelihood of success in males.

Earlier work on ape gestures suggested that the production of signal sequences is a sign of persistence in reaching a goal in response to the recipient lack of responsiveness^44^,^45^ and an attempt to increase success. In our study this was not the case, and particularly for males, signal sequences reduced the likelihood of success. For multi-modal combinations, we found a tendency to increase the likelihood of success (females and males), in concordance with previous studies showing that multi-modal signals are more efficient than single signals^40^,^51^,^52^,^53^. Overall, however, our results are more consistent with the hypothesis that multi-modal signal production serves to communicate the signaller’s social goal. Similarly, in a recent study we found that the ‘contest hoots’ of male bonobos were not more successful in triggering responses when combined with gestures^39^, but the gestures provided additional cues concerning the nature of the desired interaction^39^.

We interpret these results as indicative that signalling does not primarily function to persuade a sexual partner but to clarify the goals of the signaller. Although no clear and simple ‘production rules’ became apparent, we found that general signal categories (i.e. vocalisation, gesture, multi-modal and signal sequences) and some specific signals (i.e. ‘hand reach’, ‘scream’ and ‘pout moan’) were used in function-specific ways.

### Functional specificity of gestures and vocalisations

Our results are also in concordance with a general pattern in the animal communication literature, namely that primate vocalisations are generally more tightly linked to specific contexts than gestures^54^,^55^. In our study, we were able to identify five gestures (‘arm up’, ‘arm reach’, ‘hand reach’, ‘stretch over’ and ‘touch’) and two vocalisations (‘scream’ and ‘pout moan’) used frequently in the context of sexual solicitations. Generally, the gestures (‘arm up’, ‘arm reach’, ‘stretch over’ and ‘touch’) were used more flexibly than the two vocalisations. However, ‘hand reach’ was specifically linked to sex initiations for appeasement (for females), suggesting that primate gestures can be as functionally specific as vocalisations^56^,^57^, in line with previous work showing that primate gestures can have specific meaning^32^,^58^ or can be used to clarify the signaller’s intention in ambiguous situations^39^.

In bonobos, sexual behaviour has become largely decoupled from its original reproductive function with a diversification into the social domain and, with this study, we have demonstrated a corresponding diversification in their communicative behaviour. Our findings thus indicate a general trend in evolution, showing that complex and flexible communication can evolve from basic, fixed, and evolutionary old biological functions, a process that is characterised by increased accessibility to higher cognitive processes. To our knowledge, this study provides some of the first evidence that exemplifies this evolutionary pattern in a primate species that is of special interest, both as the closest living relative to humans and a model of the ancestral pre-human condition prior to language.

## Additional Information

**How to cite this article**: Genty, E. *et al.* Complex patterns of signalling to convey different social goals of sex in bonobos, *Pan paniscus*. *Sci. Rep.* **5**, 16135; 10.1038/srep16135 (2015).

## Supplementary Material

Supplementary Information

## Figures and Tables

**Table 1:** Results of Model 1a (females).

	b	se	*z*	P
Intercept	−1.31	1.04		
General signal type (Gesture)				
Multi-modal	−2.45	0.34		
Sequence	−1.24	0.28		
Vocalisation	−0.97	0.27		
Function (Tension reduction)				
Appeasement	0.21	0.59		
Bonding	−1.33	0.52		
Distance	−0.34	0.13	−2.55	0.0109
Relative Rank (Equal)				
Signaler is higher	0.71	1.05	0.67	0.5006
Recipient is higher	0.57	0.66	0.87	0.3835
Signaler age (Adult)				
Non-adult	0.04	0.97	0.04	0.9693
Recipient age (Adult)				
Non-adult	−0.02	0.49	−0.05	0.9632
Recipient sex (Female)				
Male	−0.12	0.44	−0.28	0.7762
Interaction signal type * function (Gesture : Tension reduction)				
Multi-modal: Appeasement	0.32	0.73	0.44	0.6622
Sequence: Appeasement	1.24	0.61	2.05	0.0405
Vocalisation: Appeasement	1.40	0.60	2.32	0.0205
Multi-modal: Social Bonding	1.51	0.53	2.84	0.0045
Sequence: Social Bonding	0.40	0.50	0.81	0.4168
Vocalisation: Social Bonding	2.52	0.50	5.05	0.0000

The data set comprised 292 initiations by 17 females. For intercept and terms comprised in the interaction, test statistics and P values are omitted. Reference levels of factors are given in parentheses.

**Table 2:** Results of Model 1b (males).

	β	Se	*z*	P
Intercept	−0.42	0.77		
General signal type (Gesture)				
Multi-modal	−0.84	0.66		
Sequence	−0.60	0.64		
Vocalisation	1.56	0.58		
Function (Appeasement)				
Reproduction	1.39	0.51		
Tension reduction	0.54	0.52		
Distance	−0.20	0.08	−2.54	0.0110
Relative Rank (Equal)				
Signaler is higher	−0.74	0.33	−2.26	0.0239
Recipient is higher	−0.89	0.39	−2.32	0.0204
Signaler age (Adult)				
Non-adult	−0.33	0.53	−0.62	0.5364
Recipient age (Adult)				
Non-adult	−0.53	0.35	−1.49	0.1353
Recipient sex (Female)				
Male	0.83	0.30	2.78	0.0054
Interaction signal type * function (Gesture : Appeasement)				
Multi-modal: Reproduction	−2.13	0.69	−3.08	0.0021
Sequence: Reproduction	−1.08	0.66	−1.64	0.1008
Vocalisation: Reproduction	−3.30	0.61	−5.40	0.0000
Multi-modal: Tension reduction	−1.76	0.76	−2.30	0.0213
Sequence: Tension reduction	−0.04	0.69	−0.06	0.9557
Vocalisation: Tension reduction	−2.40	0.64	−3.74	0.0002

The data set comprised 719 initiations by 19 males. For intercept and terms comprised in the interaction, test statistics and P values are omitted. Reference levels of factors are given in parentheses.

**Table 3:** Results of model 2a (females).

	β	se	*z*	P
Intercept	−1.67	1.13		
Social goal (Appeasement)				
Reproduction	0.23	0.69	0.34	0.7364
Tension reduction	1.61	0.58	2.77	0.0056
Gestures (Absent)				
present	0.10	0.34	0.31	0.7545
Vocalisations (Absent)				
present	−1.80	0.81	−2.23	0.0260
Sequence (No)				
yes	−0.37	0.37	−1.00	0.3153
Multi-modal (No)				
yes	1.33	0.82	1.62	0.1045
Distance	−0.28	0.16	−1.77	0.0768
Relative Rank (Equal)				
Signaler is higher	0.26	0.94	0.28	0.7805
Recipient is higher	−0.98	0.79	−1.24	0.2138
Signaler age (Adult)				
Non-adult	0.85	0.63	1.35	0.1763
Recipient age (Adult)				
Non-adult	1.30	0.46	2.83	0.0047
Recipient sex (Female)				
Male	−1.25	0.49	−2.56	0.0104

The data set comprised 292 initiations by 17 females. Test statistics and P values are omitted for the intercept. Reference levels of factors are given in parentheses.

**Table 4:** Results from Model 2b (males).

	β	se	*z*	P
Intercept	0.01	0.92		
Social goal (Appeasement)				
Reproduction	−0.16	0.50	−0.32	0.7489
Tension reduction	−1.07	0.48	−2.26	0.0239
Gestures (Absent)				
present	0.31	0.22	1.42	0.1558
Vocalisations (Absent)				
present	−1.07	0.78	−1.37	0.1709
Sequence (No)				
yes	−0.66	0.25	−2.70	0.0070
Multi-modal (No)				
yes	1.19	0.74	1.59	0.1111
Distance	−0.30	0.11	−2.79	0.0052
Relative Rank (Equal)				
Signaler is higher	−0.02	0.48	−0.04	0.9663
Recipient is higher	0.34	0.59	0.58	0.5599
Signaler age (Adult)				
Non-adult	−0.19	0.57	−0.33	0.7408
Recipient age (Adult)				
Non-adult	1.09	0.68	1.61	0.1076
Recipient sex (Female)				
Male	0.45	0.59	0.76	0.4452

The data set comprised 719 initiations by 19 males. Test statistics and P values are omitted for the intercept. Reference levels of factors are given in parentheses.
