# Supplementary material for: Complex patterns of signalling to convey different social goals of sex in bonobos, Pan paniscus
Source: Sci Rep. 2015 Nov 5;5:16135. doi: 10.1038/srep16135 (PMC4633609; doi:10.1038/srep16135)
Supplement: Supplementary Information [file srep16135-s1.pdf]

Complex patterns of signalling to convey different social goals of sex in bonobos, *Pan paniscus*

Emilie Genty[a,*], Christof Neumann[a], and Klaus Zuberbühler[a, b]

Authors affiliation: [a] Department of Comparative Cognition, Institute of Biology, University of Neuchâtel, rue Emile Argand 11, 2000 Neuchâtel, Switzerland. [b] School of Psychology and Neuroscience, University of St Andrews, St Andrews, KY16 9JP, Scotland (UK)

***Corresponding author:** Emilie Genty, Department of Comparative Cognition, Institute of Biology, University of Neuchâtel, rue Emile Argand 11, 2000 Neuchâtel, Switzerland, +41 (0) 32 718 31 15, emilie.genty@unine.ch.

Supplementary information

Supplementary methods

Statistical analysis

To analyse the signalling behaviour of bonobos during sexual initiations, our strategy went as follows. First, we aimed at testing for differences in signalling between the different social goals of sexual initiations. Because of the large number of signals used by bonobos, we started by looking at more general usage, i.e. we lumped signals into larger categories (vocalisations, gestures, multi-modal combinations and sequences of signals). Based on these results, we then investigated the use of specific signals by looking at the proportion of use of the most common signals (i.e. signals that occurred in at least 10% of initiations in at least one sex-goal combination) and suggest a set of "rules" or strategies that bonobos could employ during signalling to initiate sexual interactions. Finally, we looked at whether these rules worked in predicting success of initiations.

We ran generalized linear mixed models[1,2] separately for female and male initiators because the goals for which female and male bonobos initiated sexual interactions differed between sexes.

We considered the following variables in our models:

- Social goal: categorical, four levels: tension reduction (both sexes), appeasement (both sexes), social bonding (females only), and reproduction (males only)
- General signal type:
    - Gesture (categorical, two levels: yes/no)
    - Vocalisation (categorical, two levels: yes/no)
    - Multi-modal (categorical, two levels: yes/no)
    - Sequence of signals (categorical, two levels: yes/no)
- Recipient sex (categorical, two levels: male, female), and age (categorical, two levels: non-adult, adult)
- Signaller age (categorical, two levels: non-adult, adult)
- Relative rank (categorical, three levels: signaller higher, lower, or equal to recipient)

- Distance between signaller and recipient (continuous)

Model 1a and 1b ("general models")

With these models we aimed at testing whether different general types of signals (vocalisation, gesture, multi-modal combination, sequence) were differently likely to occur depending on the initiator's social goal. Because we were primarily interested in differences between goals, we restructured our data set in the following way (separately for females and males): for each event/initiation we created three additional lines in our data set and assigned each of the now four lines per initiation event one of the four possible signal types. The observed combination(s) of signal type and goal for a given event then received a "1" in our response variable, i.e. they occurred, while the remaining (non-observed) signal types were recorded as "0", i.e. they did not occur. Note that in this way a given event could contain occurrences for all or none of the four signal types, or any combination of signal types. This approach allowed us to test the effect of the interaction between social goal and signal types on the likelihood of their occurrence. In other words, we were able to gauge whether certain signal categories were more likely to occur in with respect to one goal as compared to another. Other potentially meaningful interactions could have been imagined, for instance between distance and signal type. We refrained from testing these because of the danger of over-fitting due to including too many parameters in the model. The female data set comprised 1,168 lines representing 292 signalling events from 17 females. The male data set comprised 2,876 lines representing 719 signalling events from 19 males.

Our initial full model contained as predictor variables the two-way interaction between goal and signal type, and the following main effects: signaller age, recipient age and sex, relative rank, and distance between signaller and recipient. In addition, our models contained random effects for signaller ID, recipient ID as well as for the event (given that we had four lines per event in the models). We tested the significance of the interaction between goal and signal type by means of a likelihood ratio test (LRT) [3]. Similarly, we tested full models versus null models with LRTs.

We tested for collinearity using the function vif of the car package[4] to calculate variance inflation factors (VIF), applied on general linear models excluding random effects and interaction terms. All VIFs were smaller than 2.0, indicating that the collinearity between our predictor variables was not a concern [3,5]. We did not detect obvious violations of the assumptions for our models. All models were fitted with the glmer function (binomial error function and logit link) of the lme4 package (version 1.1-7)[6] in R 3.1.1 [7].

Model 2a and 2b (“success models”)

Here we tested whether signal types used in initiations were predictive of accomplishing successful sexual interactions. In contrast to the models described above, we used the four signal types as separate categorical predictor variables (coded as having occurred or not) to model whether an initiation was successful or not. Each initiation event was represented as one line in our data set (292 initiation events by female initiators and 719 events by male initiators). In our full models we initially also included the two-way interactions between social goal and the four signal types. We also included the same control predictors as in the previous models. Like above, we did not include other potentially meaningful interactions to avoid over-fitting. Significance of interactions was determined with LRTs. Interactions were removed if this test did not reach significance at $P < 0.05$ to allow interpretation of the respective main effects, which is not possible otherwise[8,9].

We built these models with the same specifications as above. The largest VIF was 3.41.

Supplementary figures

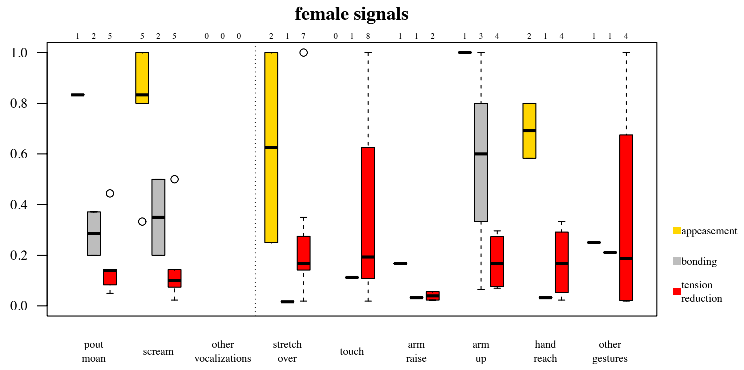

Figure S1. Proportions of usage of specific vocal and gestural signals across the different social goals of female sexual initiations. For comparison, all remaining vocalisations and gestures were lumped into one category each (other vocalisations and other gestures). Depicted are median (thick line), quartiles (box), and 1.5 * interquartile range (whiskers). Sample sizes ($n_{females}$) are indicated above each boxplot.

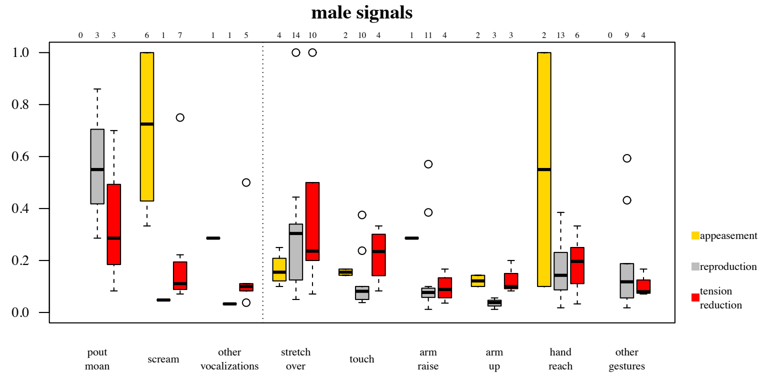

Figure S2. Proportions of usage of specific vocal and gestural signals across the different social goals of male sexual initiations. For comparison, all remaining vocalisations and gestures are lumped into one category each (other vocalisations and other gestures). Depicted are median (thick line), quartiles (box), and 1.5 * interquartile range (whiskers). Sample sizes (n_{males}) are indicated above each boxplot.

References

1. Baayen, R. H. *Analyzing Linguistic Data: A Practical Introduction to Statistics using R*. (Cambridge University Press, 2008).
2. Bolker, B. M. *et al.* Generalized linear mixed models: a practical guide for ecology and evolution. *Trends Ecol. Evol.* **24,** 127–135 (2009).
3. Quinn, G. P. & Keough, M. J. *Experimental Design and Data Analysis for Biologists*. (Cambridge University Press, 2002).
4. Fox, J. & Weisberg, S. *An R Companion to Applied Regression*. (Sage, 2011).

5. Field, A., Miles, J. & Field, Z. *Discovering Statistics Using R*. (Sage Publications, 2012).
6. Bates, D., Maechler, M., Bolker, B. & Walker, S. *lme4: Linear mixed-effects models using Eigen and S4, version* 1.1-7*, http://CRAN.R-project.org/package=lme4*. (2014).
7. R Core Team. *R: A Language and Environment for Statistical Computing*. (2014).
8. Hector, A., von Felten, S. & Schmid, B. Analysis of variance with unbalanced data: an update for ecology & evolution. *J. Anim. Ecol.* **79,** 308–316 (2010).
9. Mundry, R. Issues in information theory-based statistical inference—a commentary from a frequentist's perspective. *Behav. Ecol. Sociobiol.* **65,** 57–68 (2011).
